# Prediction of Left Ventricle Pressure Indices Via a Machine Learning Approach Combining ECG, Pulse Oximetry, and Cardiac Sounds: a Preclinical Feasibility Study

**DOI:** 10.1007/s12265-024-10546-2

**Published:** 2024-07-17

**Authors:** Lorenzo Fassina, Francesco Paolo Lo Muzio, Leonhard Berboth, Jens Ötvös, Alessandro Faragli, Alessio Alogna

**Affiliations:** 1 Department of Electrical, Computer and Biomedical Engineering, University of Pavia, Via Ferrata 5, Pavia, 27100 Italy; 2 Department of Cardiology, Deutsches Herzzentrum Der Charité, Angiology and Intensive Care Medicine, Campus Virchow-Klinikum, Augustenburgerplatz 1, Berlin, 13353 Germany; 3 DZHK (German Centre for Cardiovascular Research), Partner Site, Berlin, 10785 Germany

**Keywords:** Heart Failure, HFrEF, Porcine animal model, Invasive hemodynamics, Electronic stethoscope, Machine Learning, Linear regression

## Abstract

Heart failure (HF) is defined as the inability of the heart to meet body oxygen demand requiring an elevation in left ventricular filling pressures (LVP) to compensate. LVP increase can be assessed in the cardiac catheterization laboratory, but this procedure is invasive and time-consuming to the extent that physicians rather rely on non-invasive diagnostic tools. In this work, we assess the feasibility to develop a novel machine-learning (ML) approach to predict clinically relevant LVP indices. Synchronized invasive (pressure–volume tracings) and non-invasive signals (ECG, pulse oximetry, and cardiac sounds) were collected from anesthetized, closed-chest Göttingen minipigs. Animals were either healthy or had HF with reduced ejection fraction and *circa* 500 heartbeats were included in the analysis for each animal. The ML algorithm showed excellent prediction of LVP indices estimating, for instance, the end-diastolic pressure with a R^2^ of 0.955. This novel ML algorithm could assist clinicians in the care of HF patients.

**Graphical Abstract:**

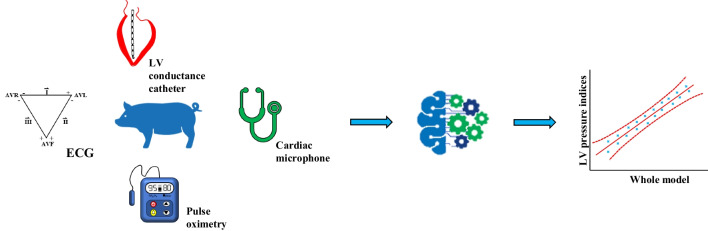

## Introduction

Heart failure (HF) is defined as the inability of the heart to meet body oxygen demand at either rest or exercise requiring an increase of cardiac filling pressure in order to meet that necessity [1]. It is a clinical syndrome with symptoms or signs caused by structural and functional cardiac abnormalities confirmed by elevated natriuretic peptide levels and/or objective evidence of pulmonary or systemic congestion [2]. Central to diagnosis are also specific thresholds of ventricular function such as left ventricular ejection fraction (LVEF).

Based on the latter parameter, HF has been subclassified into 3 categories: HF with preserved ejection fraction (LVEF ≥ 50%), HF with midrange ejection fraction (LVEF 41%-49%) and HF with reduced ejection fraction (HFrEF, LVEF < 40%) [3]. However, classifying HF on the basis of EF alone has its limitations as this parameters mostly reflects LV geometry rather than systolic function [4] and, by itself, cannot recapitulate the underlying pathophysiology of the disease [5]. Therefore, LV filling pressures plays a central role in the definition of the pathology (LV end-diastolic pressure, LV EDP > 15 mmHg at rest as a diagnostic cutoff for HF [6]), but they can only be reliably assessed via cardiac catheterization, which is invasive, time-consuming, associated with patient’s pain, and periprocedural complications [7, 8].

Regarding the bedside clinical examination, physicians rather rely on a combination of non-invasive assessments of cardiac function. However, available non-invasive methodologies such as ECG, pulse oximetry, cardiac sounds, or ultrasound imaging, have their own technical limitations [9–11], thus making the pursue of new approaches highly desirable [12].

Among the innovative approaches, a promising niche belongs to artificial intelligence (AI) who has the potential to assist clinicians in the decision-making process. Much of its merit is related to its ability to handle a vast amount of complex data and then derive novel and useful insights into clinical practice. Machine learning (ML) is an AI technique which can be designed to process data that it can quickly learn from [13]. To highlight its relevance, for instance, the U.S.A. Food and Drug Administration issued the AI/ML Action Plan (https://www.fda.gov/medical-devices/software-medical-device-samd/artificial-intelligence-and-machine-learning-software-medical-device), which aims to integrate ML and AI into medical device software.

In this work, we set out to investigate the feasibility of a ML approach to non-invasively predict clinically relevant hemodynamic indices of LV function such as: end-diastolic pressure (EDP), end-systolic pressure (ESP), and maximum rate of pressure rise (dPdt_max_). In order to build up our ML model, we have invasively measured the above-mentioned hemodynamic indices in a clinically relevant, tachypacing-induced, porcine model of HFrEF [14] as well as in a healthy control. ML features (i.e. predictors) were extracted from simultaneously acquired non-invasive signals such as ECG, pulse oximetry and cardiac sounds.

## Materials and Methods

### Porcine Model of Heart Failure with Reduced Ejection Fraction

The experimental protocol was approved by the local bioethics committee of Berlin, Germany (G 0064/19), and conforms to the Guide for the Care and Use of Laboratory Animals published by the US National Institute of Health (NIH Publication No. 85–23, revised 1996). The 2 animals included in the present proof-of-concept work were part of an already published study in which HF was induced in minipigs (Ellegaard Göttingen Minipigs, Dalmose, DK) by continuous right ventricular (RV) pacemaker stimulation (tachypacing) [14, 15]. Briefly, minipigs were exposed to incremental ventricular tachycardia for a total of 6 weeks, with 180 beats/min for 2 weeks followed by 200 beats/min for further 4 weeks. Already after 4 weeks of pacing, chronic compensated HF with highly depressed LVEF (< 40%) was induced in all animals, with an average of 22 ± 6% at 6 weeks. Healthy Göttingen minipigs were enrolled as control group [15]. At 6 weeks, final invasive LV pressure–volume measurements and right heart catheterization were performed. Animals were acutely instrumented closed-chest under fluoroscopic guidance with a pulmonary artery flotation catheter (Swan Ganz, paediatric, 5F, Edwards Lifesciences connected to Vigilance II, Edwards Lifesciences, Irvine, CA) and a LV conductance catheter (5F, 12 electrodes, 7-mm spacing; MPVS Ultra, Millar Instruments, Houston, TX) as previously described [16].

### Acquisition of Signals

In the final invasive measurement, the following signals were acquired via LabChart 8 software (ADInstruments Ltd, Oxford, UK) at a sampling frequency of 1 kHz in anesthetized, closed-chest, Göttingen minipigs:

- 1) ECG signal [V], using a standard 3-lead ECG;
- 2) LVP signal [mmHg], which is the LV pressure derived by the conductance catheter fluoroscopically positioned into the LV apex;
- 3) MIC signal [mmHg], namely, the phonocardiogram obtained via a microphone (MLT201 Cardio Microphone, ADInstruments) attached to the thoracic skin at the level of LV apex;
- 4) POX signal [mmHg], that is, the pulse oximetry acquired with a tail-cuff.

### In-Vivo Experimental Protocol and Preliminary Processing of the Signals

The acquisition of the signals started at spontaneous heart rate (HR), followed by incremental RV pacing rates with steps of 20 beats/min up to a maximum of 160 beats/min, for a total of 5 measurement steps (spontaneous, 100, 120, 140 and 160 beats/min). These steps are routinely performed for a complete hemodynamic assessment of LV function during progressively decreasing diastolic times in order to simulate the HR increase during exercise or stress. For further analysis, each recorded step was cut by selecting a duration of at least 3 min of stable hemodynamic conditions, i.e. with less than 10% variation in systemic pressure. In particular, using a custom-made script written in MATLAB® language (The MathWorks, Inc., Natick, MA), the HR was detected via ECG analysis, then the MIC signal was up- and down-enveloped, and the difference between the two envelopes was calculated for each cut segment (i.e. measurement step). The envelope of an oscillating signal is a smooth curve outlining its extremes, in our case the local maxima and the local minima of the MIC signal; the envelope, thus, generalizes the concept of a constant amplitude into an instantaneous amplitude. The difference between the upper and lower envelopes is called MED signal [mmHg] (Microphone Envelope Difference) and we bandpass-filtered it by passing only the HR previously detected in its specific segment. In conclusion, we have obtained five MED signals, one for each pacing step.

**Fig. 1:**
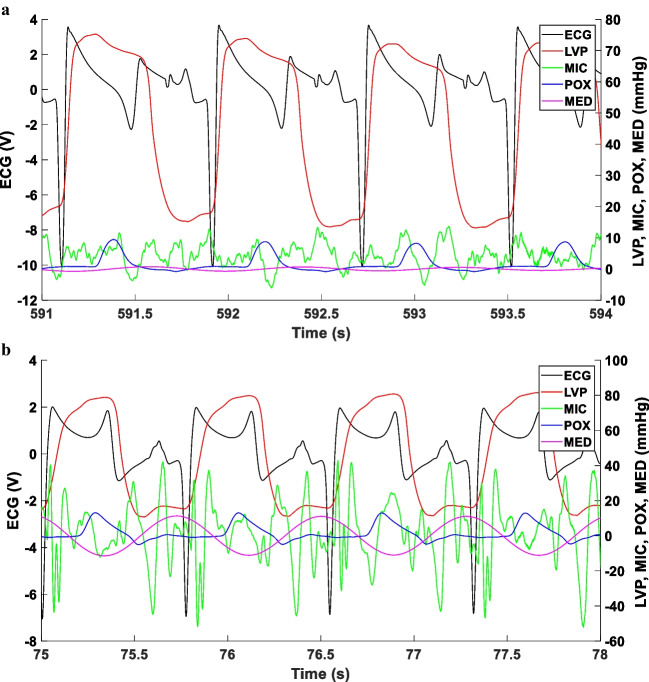
Synchronization of ECG, left ventricular pressure, cardiac sounds and pulse oximetry signals in Göttingen minipigs. Signal synchronization with ECG in black, left ventricle pressure (LVP) in red, cardiac sounds (MIC) in green, and pulse oximetry (POX) in blue. All signals were recorded, at a sampling frequency of 1 kHz, in 1 un-paced healthy swing (panel **a**) and 1 un-paced HFrEF swine (panel **b**), both with a spontaneous heart rate of circa 80 beats/min. The Microphone Envelope Difference (MED), which is the difference between the upper and lower envelopes of the MIC signals, is displayed in pink. The local minima of the MED sinus-like signal were useful, for each couple of consecutive heart cycles, to approximate the temporal boundary between systole and diastole without using the LVP signal. Note that the cardiac sounds are louder in the un-paced heart failure swine

**Fig. 2:**
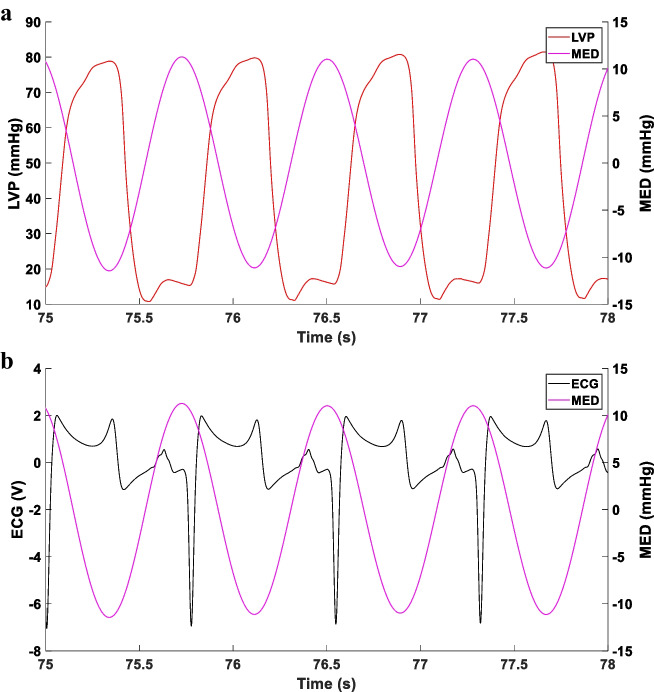
Representative Microphone Envelope Difference (MED) in an un-paced heart failure Göttingen minipig. The graphs highlight that the local minima of the MED sinus-like signal can be used to approximate the temporal boundary between systole and diastole for each couple of consecutive heart cycles (panel **a** and **b**). In detail, the local minima of the MED signal identify the peaks of the LVP signal

As displayed in Figs. 1 and 2, the local minima of the MED sinus-like signal were useful, for each couple of consecutive heart cycles, to approximate the temporal boundary between systole and diastole without using the LVP signal (Figs. 1 and 2). More specifically, the local minima of the MED sinus-like signal identify the peaks of the LVP signal and the T wave on the ECG signal.

### Features Extraction for the Prediction of Left Ventricle Indices

The main aim of our study was to define features (i.e., predictors) extracted from cardiac sounds, ECG, and pulse oximetry in order to predict via linear regression the following LV pressure indices: EDP [mmHg], ESP [mmHg], and dPdt_max_ [mmHg/s].

Using a custom-made script written in MATLAB® language, via ECG analysis and by the local minima of the MED sinus-like signal, each heartbeat was identified and segmented into diastole and systole for the single-beat analysis of the following indices:

i) EDP, ESP, dPdt_max_ (parameters to be predicted) calculated from the invasively assessed LVP signal;
ii) the multiplicative inverse of the pulse transit time (invPTT) calculated from the ECG and the pulse oximetry signals.

The invPTT has been already described in literature as a good predictor of systemic blood pressure as, for instance, Wibmer et al. [17] found a clear negative correlation between pulse transit time (PTT) and mean systolic blood pressure, as previously reviewed [18].

In addition, for each heartbeat and separately for diastole and systole, we analyzed the melody spectrum (MEL) of the MIC signal, from which we computed two sets of predictors employing the MATLAB® Audio Toolbox™ (The MathWorks, Inc., Natick, MA):

i) set A comprising spectral flux [19], spectral kurtosis [20–23], spectral skewness [20–22], spectral slope [24, 25];
ii) set B including the 13 MEL frequency cepstral coefficients in 2nd derivative (delta-delta-MFCCs) [26–30].

The rationale of calculating both time and frequency features (invPTT and MEL predictors, respectively) is that similar combinations of “time plus frequency” have been crucial to achieve improved results in works about heart sounds. As an overall strategy, the systolic sounds were employed to predict the precedent EDP and the contemporary dPdt_max_, whereas the diastolic sounds were employed to predict the precedent ESP.

## Results

In this preclinical feasibility study, *circa* 500 cardiac cycles from each minipig (1 HFrEF and 1 healthy control) were included in the final analysis. Table 1 summarizes the baseline hemodynamic characteristics of the two animals on the day of the final invasive LV pressure–volume measurements. As expected, the HFrEF animal showed a highly depressed ejection fraction (LV EF 16%), while the healthy control had a preserved LV EF of 50%. Stroke volume and cardiac output were severely depressed in HFrEF, with LV EDP being above the diagnostic cutoff for HF (> 15 mmHg).

**Table 1:** Baseline hemodynamic characteristics of the animals included in the study

Parameter at baseline	Healthy	HFrEF
HR [bpm]	78	78
SV [ml]	26.56	17.53
CO [L/min]	2.09	1.37
EF [%]	50	15.6
mean AOP [mmHg]	57	60
LV EDP [mmHg]	5.70	15.45
LV ESP [mmHg]	64.81	77.65
dPdt_max_ [mmHg/s]	1168.75	581.55

*HR* Heart rate, *bpm* beats per minute, *SV*, Stroke volume, *CO* Cardiac output, *EF* Ejection fraction, *AOP* Aortic pressure, *LV EDP* Left ventricle end-diastolic pressure, *LV ESP* Left ventricle end-systolic pressure, *dPdt*_*max*_ maximum rate of pressure rise

### Prediction of LVP Indices

**Fig. 3:**
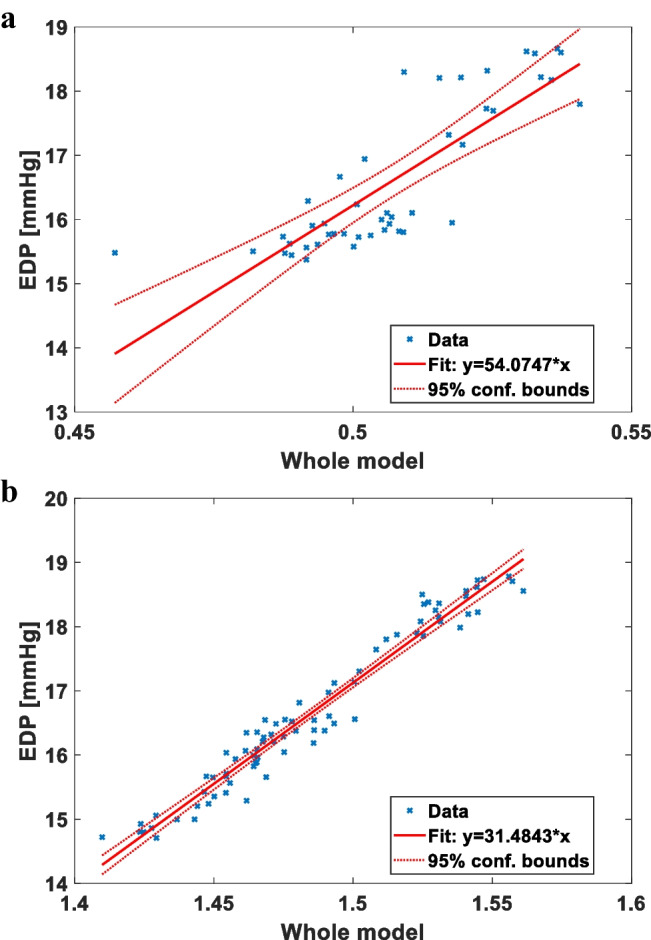
Representative linear regression analysis to predict the invasively measured LV EDP. The regressions are calculated at a pacing stimulation of 100 bpm (panel **a**, healthy animal, R^2^ = 0.694 and RMSE = 0.82 mmHg, with a relative error of less than 5%) and at a spontaneous HR of 80 bpm (panel **b**, HFrEF animal, R^2^ = 0.955 and RMSE = 0.3 mmHg, with a relative error of less than 2%). The whole model (x axis) is the linear combination of the MEL features of the cardiac sounds (set **A** and set **B**) plus the invPTT. The blue crosses represent the analyzed heartbeats, the red line is the linear regression fit to predict the LV EDP, and the red dotted curves are the 95% confidence bounds for the linear regression

In order to test the validity of the extracted features in predicting LV pressure indices, we tested the prediction of the combination of the above-described MEL features (sets A and B) with the calculated invPTT against invPTT alone. For instance, Fig. 3a shows the linear regression analysis where the “whole model” (i.e. all the predicting features condensed in their linear combination) predicts the LV EDP in a healthy animal at a pacing stimulation of 100 bpm. Figure 3b corresponds to the linear regression analysis where the whole model predicts the LV EDP in a HFrEF animal at a spontaneous HR of *circa* 80 bpm.

In particular for the HFrEF, we observed an excellent R^2^ (coefficient of determination) of 0.955 and a small RMSE (root mean square error) of *ca.* 0.3 mmHg: in other words, we committed a relative error of less than 2% in the indirect, non-invasive estimation of the LV pressure at the end of the diastole.

Table 2 summarizes the results for the prediction of LV EDP, LV ESP and LV dPdt_max_ for both the HFrEF and the healthy animals in spontaneous HR. Taken together, the MEL features of the cardiac sounds (set A plus set B) combined with the calculated invPTT showed a very good prediction skill.

**Table 2:** Results of the linear regression model for the HFrEF and healthy control Göttingen minipigs in spontaneous HR

HFrEF				
**Pacing**	**To be predicted**	**Predictors**	**R**^**2**^	**RMSE**
Spontaneous heart rate	EDP	invPTT	0.776	0.588
Spontaneous heart rate	EDP	set AB + invPTT	0.955	0.298
Spontaneous heart rate	ESP	invPTT	0.897	0.547
Spontaneous heart rate	ESP	set AB + invPTT	0.944	0.458
Spontaneous heart rate	dPdt_max_	invPTT	0.889	3.747
Spontaneous heart rate	dPdt_max_	set AB + invPTT	0.934	3.290
**Control**				
**Pacing**	**To be predicted**	**Predictors**	**R**^**2**^	**RMSE**
Spontaneous heart rate	EDP	invPTT	0.003	1.480
Spontaneous heart rate	EDP	set AB + invPTT	0.189	1.595
Spontaneous heart rate	ESP	invPTT	0.006	1.112
Spontaneous heart rate	ESP	set AB + invPTT	0.230	1.222
Spontaneous heart rate	dPdt_max_	invPTT	0.000	7.647
Spontaneous heart rate	dPdt_max_	set AB + invPTT	0.340	7.415

The unit of measurement for RMSE (root mean square error) is the same of the related predicted parameter. Each hemodynamic index was predicted by invPTT alone (representative of an accepted method in literature) and by the combination of invPTT and set AB (the frequency domain features from the phonocardiogram)

*bpm* beats per minute, *EDP* End-diastolic pressure, *ESP* End-systolic pressure, *dPdt*_*max*_ maximum rate of pressure rise, *invPTT* inverse of pulse transit time, *set AB* set of MEL predictors

Overall, the linear combination of MEL features of the cardiac sounds with the invPTT performed better in predicting the invasively measured LV pressure indices compared to invPTT alone in both healthy (LV EDP R^2^ 0.189 *vs* 0.003, LV ESP R^2^ 0.230 *vs* 0.006, LV dPdt_max_ R^2^ 0.340 *vs* 0.000; MEL + invPTT *vs* invPTT, respectively) and HFrEF pigs (LV EDP R^2^ 0.955 *vs* 0.776; LV ESP R^2^ 0.944 *vs* 0.897; LV dPdt_max_ R^2^ 0.934 *vs* 0.889; MEL + invPTT *vs* invPTT, respectively).

### Prediction of LVP Indices During the RV Pacing Steps

Tables 3 and 4 display the prediction skill of our linear regression model during RV pacing up to 160 beats/min for both HFrEF and healthy pigs. The RV pacing steps are routinely performed for the hemodynamic assessment of LV function during progressively decreasing diastolic times. Overall, the performance of our model to predict LVP indices was worsened by pacing at higher heart rates, in particular at a pacing rate of 140 bpm, probably because of the progressive shortening of the diastolic time. As it occurred during spontaneous beating, the combined use of MEL features of the cardiac sounds (set A plus set B) with the invPTT performed better than using invPTT alone.

**Table 3:** Results of the linear regression model for the HFrEF Göttingen minipig

HFrEF				
**Pacing**	**To be predicted**	**Predictors**	**R**^**2**^	**RMSE**
100 bpm	EDP	invPTT	0.379	0.539
100 bpm	EDP	set AB + invPTT	0.747	0.379
120 bpm	EDP	invPTT	0.485	0.415
120 bpm	EDP	set AB + invPTT	0.724	0.329
140 bpm	EDP	invPTT	0.066	1.308
140 bpm	EDP	set AB + invPTT	0.576	0.945
160 bpm	EDP	invPTT	0.310	2.928
160 bpm	EDP	set AB + invPTT	0.849	1.455
100 bpm	ESP	invPTT	0.863	0.747
100 bpm	ESP	set AB + invPTT	0.912	0.657
120 bpm	ESP	invPTT	0.854	0.596
120 bpm	ESP	set AB + invPTT	0.912	0.502
140 bpm	ESP	invPTT	0.105	4.589
140 bpm	ESP	set AB + invPTT	0.363	4.102
160 bpm	ESP	invPTT	0.893	1.674
160 bpm	ESP	set AB + invPTT	0.918	1.804
100 bpm	dPdt_max_	invPTT	0.859	8.175
100 bpm	dPdt_max_	set AB + invPTT	0.942	5.760
120 bpm	dPdt_max_	invPTT	0.843	10.905
120 bpm	dPdt_max_	set AB + invPTT	0.933	7.736
140 bpm	dPdt_max_	invPTT	0.053	117.579
140 bpm	dPdt_max_	set AB + invPTT	0.797	58.025
160 bpm	dPdt_max_	invPTT	0.900	45.350
160 bpm	dPdt_max_	set AB + invPTT	0.954	32.842

The unit of measurement for RMSE (root mean square error) is the same of the related predicted parameter. For each frequency step, each hemodynamic index was predicted by invPTT alone (representative of an accepted method in literature) and by the combination of invPTT and set AB (the frequency domain features from the phonocardiogram)

*bpm* beats per minute, *EDP* End-diastolic pressure, *ESP* End-systolic pressure, *dPdt*_*max*_ maximum rate of pressure rise, *invPTT* inverse of pulse transit time, *set AB* set of MEL predictors

**Table 4:** Results of the linear regression model for the healthy control Göttingen minipig

Control				
**Pacing**	**To be predicted**	**Predictors**	**R**^**2**^	**RMSE**
100 bpm	EDP	invPTT	0.497	0.801
100 bpm	EDP	set AB + invPTT	0.694	0.820
120 bpm	EDP	invPTT	0.002	1.139
120 bpm	EDP	set AB + invPTT	0.690	0.699
140 bpm	EDP	invPTT	0.547	0.961
140 bpm	EDP	set AB + invPTT	0.900	0.580
160 bpm	EDP	invPTT	0.131	4.640
160 bpm	EDP	set AB + invPTT	not available	
100 bpm	ESP	invPTT	0.448	0.830
100 bpm	ESP	set AB + invPTT	0.806	0.661
120 bpm	ESP	invPTT	0.017	0.932
120 bpm	ESP	set AB + invPTT	0.230	0.907
140 bpm	ESP	invPTT	0.744	0.498
140 bpm	ESP	set AB + invPTT	0.884	0.425
160 bpm	ESP	invPTT	0.823	2.736
160 bpm	ESP	set AB + invPTT	not available	
100 bpm	dPdt_max_	invPTT	0.000	130.251
100 bpm	dPdt_max_	set AB + invPTT	0.512	11.648
120 bpm	dPdt_max_	invPTT	0.026	29.865
120 bpm	dPdt_max_	set AB + invPTT	0.249	28.846
140 bpm	dPdt_max_	invPTT	0.371	26.943
140 bpm	dPdt_max_	set AB + invPTT	0.803	19.249
160 bpm	dPdt_max_	invPTT	0.969	52.621
160 bpm	dPdt_max_	set AB + invPTT	not available	

The unit of measurement for RMSE (root mean square error) is the same of the related predicted parameter. For each frequency step, each hemodynamic index was predicted by invPTT alone (representative of an accepted method in literature) and by the combination of invPTT and set AB (the frequency domain features from the phonocardiogram)

*bpm* beats per minute, *EDP* End-diastolic pressure, *ESP* End-systolic pressure, *dPdt*_*max*_ maximum rate of pressure rise, *invPTT* inverse of pulse transit time, *set AB* set of MEL predictors

## Discussion

This study represents a proof-of-concept aiming to assess the feasibility of a ML model to predict physiologically relevant indices of LV pressure by employing a combination of non-invasive, inexpensive, and routinely applied clinical methods. Specifically, synchronized invasive (LV pressure–volume signals) and non-invasive signals (ECG, pulse oximetry and phonocardiogram via cardiac microphone) were collected from anesthetized, closed-chest Göttingen minipigs. Animals were either healthy or had HF with reduced ejection fraction induced via right ventricular tachypacing over 6 weeks. A MATLAB® environment was used to extract a set of features from cardiac sounds, ECG, and pulse oximetry signals in order to predict the LV EDP, ESP, and dPdt_max_ via linear regression analysis. A total of *circa* 500 heart cycles per animal were included in the analysis. The developed model was able to accurately predict, for instance in the HFrEF animal, the invasively measured LV EDP with a R^2^ of 0.955 and root mean square error of 0.3 mmHg, namely, with a relative error of less than 2% in the indirect estimation of LV EDP. [31].

### Role of Left Ventricular Filling Pressure Assessment in Heart Failure

The assessment of increased LV filling pressure plays a central role in the diagnosis of HF, and LV EDP has been shown to be an important prognostic predictor in chronic stable HF patients [6]. Unfortunately, LV EDP can only be reliably measured by cardiac catheterization, which is invasive, time-consuming, and associated with risk of infection, blood clotting or embolism. Hence, clinicians seek to use non-invasive assessments such as ultrasound imaging or circulating biomarkers, which are not exempted from numerous technical limitations [12]. Therefore, a non-invasive, inexpensive, and rapid estimation of the LV filling pressure, as the one reported in this work, would be beneficial for the patient’s care. Contrary to other studies exploiting the phonocardiogram [32–34], a strength of our work consisted in using catheterization, being the gold standard for pressure assessment [4], in the development of the machine learning approach.

### Animal Models of Heart Failure and Hemodynamic Derangements

In the past decades, several animal models recapitulating the clinical phenotype of HFrEF patients’ subgroups have been established [35]. In particular, the myocardial infarction and chronic rapid pacing models are most commonly reported in literature. In the first model, an acute myocardial ischemia is induced and is expected to lead to chronic HF over time. For instance, it can be achieved by coronary artery ligation or microembolization. This approach has the benefit to model one of the most frequent underlying causes of HFrEF, but comes with risks of lethal arrhythmias, low reproducibility and often leads to moderate LV dysfunction [35]. The second model has been first described in the 1970s [36] and is based on pacing the heart at a rate 2–4 fold higher than physiological HR. Since then, it has become the gold standard of HFrEF with loss of intrinsic myocardial contractility, dilated ventricles, and reduced contractile reserve to inotropic challenge [14]. This model offers the advantage of high reproducibility and strong LV dysfunction and comes with the typical hemodynamic and neurohumoral alterations observed in HF patients [35]. Therefore, in the past decades, it has been employed in numerous translational studies and also recently by our group to investigate HF development and novel therapeutic approaches [15].

### Machine Learning to Predict Left Ventricular Pressure Indices

Our ML approach employs the linear regression model, in which LV EDP, ESP, and dPdt_max_ are the dependent variables to be predicted, and a set of 18 features, calculated from the ECG, POX, and MIC signals, are the independent variables. In detail, one feature is the multiplicative inverse of pulse transit time (invPTT), which was calculated from the ECG and POX signals. This parameter is considered a good predictor of systemic blood pressure as, for instance, Wibmer et al. [17] found a clear negative correlation between PTT and the systemic blood pressure. The other 17 features were calculated from the MEL spectrum of the MIC signal.

In the reported model of HFrEF, our ML approach produced worse predictions when invPTT has been used alone. This outcome is in line with recent evidence suggesting that combinations of temporal and frequency features (invPTT and MEL predictors, respectively) have improved the results in studies employing the heart sounds [28–30]. Indeed, we have achieved a far better prediction using the set of 18 features at each step of our frequency–response protocol for both healthy and unhealthy animals (Tables 2, 3, and 4).

### Limitations

As a major limitation to the study, we recorded the required signals for the development of the ML approach in only 2 animals that were part of a trial with already extensive investigations. However, our ML model was built to predict the LV hemodynamic indices for each cardiac cycle (*circa* 500 heartbeats analyzed per animal) and independently from the health status of the subject. As this might help with the issue of sample size, it also poses a problem in the variability of our dataset as the physiological beat-to-beat variability might be limited. Therefore, a larger study to confirm this initial proof-of-concept is warranted. Finally, these experiments were performed under anaesthesia, whereas, hypothesizing its application in the clinic, the ML algorithm will be applied in chronic stable HF patients.

## Conclusions

In summary, the newly developed machine-learning approach allows for excellent prediction of LV pressure indices. This algorithm could significantly advance both diagnostics and care of HF patients, allowing monitoring and therapy titration according to the non-invasively measured LV end-diastolic pressure.

## Data Availability

Data can be provided upon formal request.

## Abbreviations

HF: Heart Failure
LVEF: Left Ventricular Ejection Fraction
HFrEF: Heart Failure with reduced Ejection Fraction
AI: Artificial Intelligence
ML: Machine Learning
EDP: End-Diastolic Pressure
ESP: End-Systolic Pressure
dPdt_max_: Maximum rate of pressure rise
RV: Right Ventricle
LVP: Left Ventricular Pressure signal
MIC: Microphone signal
POX: Pulse Oximetry signal
HR: Heart Rate
MED: Microphone Envelope Difference signal
invPTT: Inverse of Pulse Transit Time
MEL: Melody spectrum

## References

[1] McDonagh TA, et al. 2021 ESC Guidelines for the diagnosis and treatment of acute and chronic heart failure. Eur Heart J. 2021;42(36):3599–726. 34447992 10.1093/eurheartj/ehab368

[2] Murphy SP, Ibrahim NE, Januzzi JL Jr. Heart Failure With Reduced Ejection Fraction: A Review. JAMA. 2020;324(5):488–504. 32749493 10.1001/jama.2020.10262

[3] Bozkurt B, et al. Universal Definition and Classification of Heart Failure: A Report of the Heart Failure Society of America, Heart Failure Association of the European Society of Cardiology, Japanese Heart Failure Society and Writing Committee of the Universal Definition of Heart Failure. J Card Fail. 2021;27:387–413. 10.1016/j.cardfail.2021.01.022 33663906

[4] Abawi D, et al. The non-invasive assessment of myocardial work by pressure-strain analysis: clinical applications. Heart Fail Rev. 2022;27(4):1261–79. 34041679 10.1007/s10741-021-10119-4 PMC9197903

[5] Lam CSP, Yancy C. Universal Definition and Classification of Heart Failure: Is It universal? Does It Define Heart Failure? J Card Fail. 2021;27(5):509–11. 33737145 10.1016/j.cardfail.2021.03.003

[6] Reddy YNV, El-Sabbagh A, Nishimura RA. Comparing Pulmonary Arterial Wedge Pressure and Left Ventricular End Diastolic Pressure for Assessment of Left-Sided Filling Pressures. JAMA Cardiol. 2018;3(6):453–4. 29590308 10.1001/jamacardio.2018.0318

[7] Manda YR, Baradhi KM. Cardiac catheterization risks and complications. 2023 Jun 5. In: StatPearls [Internet]. Treasure Island (FL): StatPearls Publishing; 2024. 30285356

[8] Al-Hijji MA, et al. Safety and Risk of Major Complications With Diagnostic Cardiac Catheterization. Circ Cardiovasc Interv. 2019;12(7):e007791. 31284736 10.1161/CIRCINTERVENTIONS.119.007791

[9] Gurun Kaya A, et al. Is pulse oximeter a reliable tool for non-critically ill patients with COVID-19? Int J Clin Pract. 2021;75(12):e14983. 34637170 10.1111/ijcp.14983 PMC8646536

[10] Grant MD, et al. Transthoracic Echocardiography: Beginner’s Guide with Emphasis on Blind Spots as Identified with CT and MRI. Radiographics. 2021;41(4):1022–42. 34115535 10.1148/rg.2021200142 PMC8493765

[11] Malik SB, et al. Transthoracic Echocardiography: Pitfalls and Limitations as Delineated at Cardiac CT and MR Imaging. Radiographics. 2017;37(2):383–406. 28212053 10.1148/rg.2017160105

[12] Faragli A, et al. The role of non-invasive devices for the telemonitoring of heart failure patients. Heart Fail Rev. 2021;26(5):1063–80. 32338334 10.1007/s10741-020-09963-7 PMC8310471

[13] Sevakula RK, et al. State-of-the-Art Machine Learning Techniques Aiming to Improve Patient Outcomes Pertaining to the Cardiovascular System. J Am Heart Assoc. 2020;9(4):e013924. 32067584 10.1161/JAHA.119.013924 PMC7070211

[14] Powers JC, Recchia F. Canine Model of Pacing-Induced Heart Failure. Methods Mol Biol. 2018;1816:309–25. 29987830 10.1007/978-1-4939-8597-5_24

[15] Alogna A, et al. Lung-to-Heart Nano-in-Micro Peptide Promotes Cardiac Recovery in a Pig Model of Chronic Heart Failure. J Am Coll Cardiol. 2024;83(1):47–59. 38171710 10.1016/j.jacc.2023.10.029

[16] Alogna A, et al. Inotropic Effects of Experimental Hyperthermia and Hypothermia on Left Ventricular Function in Pigs-Comparison With Dobutamine. Crit Care Med. 2016;44(3):e158–67. 26474110 10.1097/CCM.0000000000001358

[17] Wibmer T, et al. Pulse transit time and blood pressure during cardiopulmonary exercise tests. Physiol Res. 2014;63(3):287–96. 24564606 10.33549/physiolres.932581

[18] Mukkamala R, et al. Toward Ubiquitous Blood Pressure Monitoring via Pulse Transit Time: Theory and Practice. IEEE Trans Biomed Eng. 2015;62(8):1879–901. 26057530 10.1109/TBME.2015.2441951 PMC4515215

[19] Hao Y, et al. Spectral Flux-Based Convolutional Neural Network Architecture for Speech Source Localization and Its Real-Time Implementation. IEEE Access. 2020;8:197047–58. 33981519 10.1109/access.2020.3033533 PMC8112575

[20] Tanner K, et al. Spectral moments of the long-term average spectrum: sensitive indices of voice change after therapy? J Voice. 2005;19(2):211–22. 15907436 10.1016/j.jvoice.2004.02.005

[21] Lowell SY, et al. Spectral- and cepstral-based measures during continuous speech: capacity to distinguish dysphonia and consistency within a speaker. J Voice. 2011;25(5):e223–32. 20971612 10.1016/j.jvoice.2010.06.007

[22] Colton RH, et al. Spectral moment analysis of unilateral vocal fold paralysis. J Voice. 2011;25(3):330–6. 20813498 10.1016/j.jvoice.2010.03.006

[23] Antoni J, Randall RB. The spectral kurtosis: application to the vibratory surveillance and diagnostics of rotating machines. Mech Syst Signal Process. 2006;20(2):308–31.

[24] Nakamura T, Yamauchi Y, Kawahara K. Valid method to evaluate the slope of Fourier transformed spectrum for the analysis of biological rhythm fluctuation. Biomed Mater Eng. 1995;5(1):21–8. 7773143

[25] Guzman M, et al. Comparison of Supraglottic Activity and Spectral Slope Between Theater Actors and Vocally Untrained Subjects. J Voice. 2016;30(6):767e1–8. 10.1016/j.jvoice.2015.10.017 26725552

[26] Bardou D, Zhang K, Ahmad SM. Lung sounds classification using convolutional neural networks. Artif Intell Med. 2018;88:58–69. 29724435 10.1016/j.artmed.2018.04.008

[27] Bozkurt B, Germanakis I, Stylianou Y. A study of time-frequency features for CNN-based automatic heart sound classification for pathology detection. Comput Biol Med. 2018;100:132–43. 29990646 10.1016/j.compbiomed.2018.06.026

[28] Nogueira DM, et al. Classifying Heart Sounds Using Images of Motifs, MFCC and Temporal Features. J Med Syst. 2019;43(6):168. 31056720 10.1007/s10916-019-1286-5

[29] Aziz S, et al. Phonocardiogram Signal Processing for Automatic Diagnosis of Congenital Heart Disorders through Fusion of Temporal and Cepstral Features. Sensors (Basel). 2020;20(13):3790. 32640710 10.3390/s20133790 PMC7374414

[30] Khan FA, Abid A, Khan MS. Automatic heart sound classification from segmented/unsegmented phonocardiogram signals using time and frequency features. Physiol Meas. 2020;41(5):055006. 32259811 10.1088/1361-6579/ab8770

[31] Abawi D, et al. Cardiac power output accurately reflects external cardiac work over a wide range of inotropic states in pigs. BMC Cardiovasc Disord. 2019;19(1):217. 31615415 10.1186/s12872-019-1212-2 PMC6792198

[32] Ogawa S, Namino F, Mori T, Sato G, Yamakawa T, Saito S. AI diagnosis of heart sounds differentiated with super stethoscope. J Cardiol. 2023;83(4):265–271. 10.1016/j.jjcc.2023.09.007 10.1016/j.jjcc.2023.09.007 37734656

[33] Reyna MA, et al. Heart murmur detection from phonocardiogram recordings: The George B. Moody PhysioNet Challenge 2022. PLOS Digit Health. 2023;2(9):e0000324. 37695769 10.1371/journal.pdig.0000324 PMC10495026

[34] Bachtiger P, et al. Point-of-care screening for heart failure with reduced ejection fraction using artificial intelligence during ECG-enabled stethoscope examination in London, UK: a prospective, observational, multicentre study. Lancet Digit Health. 2022;4(2):e117–25. 34998740 10.1016/S2589-7500(21)00256-9 PMC8789562

[35] Charles CJ, et al. Large Animal Models of Heart Failure: Reduced vs Preserved Ejection Fraction. Animals (Basel). 2020;10(10):1906. 33080942 10.3390/ani10101906 PMC7603281

[36] Coleman HN 3rd, et al. Congestive heart failure following chronic tachycardia. Am Heart J. 1971;81(6):790–8. 5088355 10.1016/0002-8703(71)90083-4

